# Isolation and Characterization of Commensal Bifidobacteria Strains in Gut Microbiota of Neonates Born Preterm: A Prospective Longitudinal Study

**DOI:** 10.3390/microorganisms10030654

**Published:** 2022-03-18

**Authors:** Sandra Wydau-Dematteis, Johanne Delannoy, Anne-Claire Téolis, Agnès Giuseppi, Florence Campeotto, Alexandre Lapillonne, Marie-José Butel, Julio Aires

**Affiliations:** 1 3PHM, INSERM, Faculté de Santé, Université Paris Cité, F-75006 Paris, France; johanne.delannoy@u-paris.fr (J.D.); annecteo@hotmail.com (A.-C.T.); florence.campeotto@nck.aphp.fr (F.C.); mariejobutel@gmail.com (M.-J.B.); julio.aires@u-paris.fr (J.A.); 2 Maternité Port-Royal, AP-HP, Université Paris Cité, Fédération Hospitalo-Universitaire Combattre la Prématurité, F-75006 Paris, France; 3 Service de Pédiatrie et Réanimation Néonatales, Hôpital Necker-Enfants Malades, AP-HP, F-75015 Paris, France; agnes.giuseppi@nck.aphp.fr (A.G.); alexandre.lapillonne@aphp.fr (A.L.); 4 Service de Gastroentérologie et Nutrition Pédiatriques, Hôpital Necker-Enfants Malades, AP-HP, F-75015 Paris, France; 5 EHU 7328 PACT, Université Paris Cité, F-75015 Paris, France

**Keywords:** bifidobacteria, microbiota, preterm neonate

## Abstract

Bifidobacterial population dynamics were investigated using a longitudinal analysis of dominant species isolated from feces of neonates born preterm (singletons (*n* = 10), pairs of twins (*n* = 11)) from birth up to 16 months of age. We performed quantification, isolation, and identification of the dominant bifidobacteria strains. The genetic relationship of the isolates was investigated via pulsed field gel electrophoresis (PFGE) genotyping, and PCR was used to screen the specific genetic marker *tet* genes. Additionally, all of the isolated strains were phenotypically characterized by their response to gastro-intestinal stresses and the MIC determination of tetracycline. In the same individual, our results showed a turnover of the bifidobacteria dominant population not only at species but also at strain levels. In addition, we found clonally related strains between twins. A minority of strains were tolerant to gastric (6%) and intestinal (16%) stresses. Thirteen percent of the strains were resistant to tetracycline. This work is original as it provides insights at the strain level of the early life in vivo dynamics of gut microbiota bifidobacteria in preterm neonates. It highlights the need to take into consideration the fluctuation of bifidobacteria populations that may occur for one individual.

## 1. Introduction

The genus *Bifidobacterium* belongs to the dominant gut microbiota particularly in infants [1,2], thereby implicating this bacterial group as one of the main microbial candidates that affect the physiology and immunology of a host. Hence, this genus is considered to have health benefits with claimed probiotic properties [3,4,5]. The use of molecular approaches has provided novel insights into gut microbiota analysis, showing that the intestinal microbiota community is more complex than previously described [6,7,8]. In adults, fecal microbiota has been shown to be individual-specific and represented by limited diversity at the phylum level [9,10]. In infants, the dynamic process of colonization has been well studied at high taxonomic levels [11,12], but is incomplete at lower taxonomic levels. Moreover, mounting importance is given to the first colonizing bacteria and factors leading to an early gut dysbiosis are recognized to increase the risk of developing short- and long-term diseases [13,14,15,16]. In very preterm neonates (PNs), microbiota establishment is characterized by a significant delay in the colonization of bacteria belonging to the *Bacteroides* and *Bifidobacterium* genera compared to full term neonates [17,18,19,20]. In contrast, colonization by staphylococci and the *Clostridium* genus is common [20,21,22]. In addition, abnormal early bacterial gut colonization profiles have been associated with an increased risk of necrotizing enterocolitis (NEC) or neurodevelopmental impairment [22,23,24].

Improving health in the population of neonates born prematurely is a clinical challenge. The promotion of beneficial microbiota establishment by supplementing neonates with prebiotics and/or probiotics has received increasing attention [25,26,27]. The use of probiotics in PNs has been associated with several health benefits, such as gain in body weight and reduced incidence and/or severity of NEC and of late-onset sepsis [25,26,27]. However, only conditional recommendations with a low certainty of evidence have been recently made by the Nutrition Committee of the ESPGHAN for the use of probiotics in PNs because there is still only limited evidence available [27].

Data on the kinetics of colonization by bifidobacteria in PNs are scarce. Indeed, studies previously focused on the identification and/or quantification of gut bifidobacteria colonization in relation to geographical location, age, gender or diet [1,2,28,29,30,31,32,33,34,35]. These studies, including molecular-based techniques such as 16S rRNA gene sequencing or metagenomics, provide a global and static view of the presence of bifidobacteria in gut microbiota, do not differentiate single or multiple strains, and do not allow a phenotypic validation of the genetic potential of the microbiome [2,6,19,33]. In PNs, data are even rarer, while probiotic supplementation has been proposed as a therapeutic strategy to improve the gut microbiota of this population [36,37,38]. Hence, there is a lack of basic knowledge of the bifidobacterial population dynamics that would enable us to reliably investigate the impact of dietary components such as probiotics or prebiotics on the intestinal ecosystem.

In this prospective longitudinal study, our goal was to isolate and follow up the bifidobacteria strains able to colonize infants born premature during the first year of life and compare the strains at both phenotypic and genetic levels. For this purpose, we used a culture approach that allowed for deep analysis of the strains at both phenotypic and genotypic levels.

## 2. Materials and Methods

### 2.1. Study Design

This study was performed using the bifidobacteria strains isolated from fecal samples of infants born preterm included in a prospective longitudinal study during the first year of life (PREMAFLORA-ANR-07-PNRA-007). Stool samples were collected from PNs born at less than 37 weeks of gestational age and hospitalized at a French pediatric hospital. They were collected from June 2008 to March 2009 weekly from birth to hospital discharge, and every 3 months during the first year of life after hospital discharge for 47 infants. For the current study, we included 26 neonates for whom we had at least 2 fecal samples with bifidobacterial colonization at different times. In addition, we included 3 pairs of twins who had only one fecal sample with bifidobacteria during follow-up. PNs suffering from malformations or metabolic diseases were excluded. Samples (about 1 g) were diluted in 0.5 mL of Brain Heart Infusion (BHI) broth with 15% of glycerol (cryoprotectant) and kept at −80 °C until analyses were carried out. The study was conducted in accordance with the relevant French guidelines and regulations. A written informed parental consent was obtained for each sample before inclusion (during this time period, parental consent was sufficient to ensure that fecal samples were collected under ethical conditions).

### 2.2. Bifidobacteria Strains’ Isolation, Quantification and Identification

For bifidobacteria enumeration and selection, homogenate fecal samples in pre-reduced BHI broth were diluted in peptone water (NaCl 0.85%). Serial dilutions (10^−2^, 10^−4^ and 10^−6^) were spread using a WASP apparatus (bioMerieux, Marcy-l’Etoile, France) on bifidobacteria selective WCBM media [39], and incubated for 48 h at 37 °C in an MAC500 chamber (bioMerieux) under anaerobic conditions (CO_2_:H_2_:N_2_, 10:10:80). Bifidobacteria colony counts were calculated from dilution plates on WCBM with 20 to 200 colonies and were expressed as the log_10_ CFU/g of feces. The threshold of detection was 3 log_10_ CFU/g of feces.

For each fecal sample, up to 10 random colonies suspected to be bifidobacteria (based on their ability to grow on WCBM and their cellular morphology) were chosen for analysis. It was decided to examine 10 random colonies per sample on the basis of previous reports showing that this number gave correct representation of the major bacterial strains cultured on a selective medium [40,41].

These colonies were grown for 48 h at 37 °C under anaerobic conditions in TGYH broth (Tryptone 30 g.L^−1^; Glucose 5 g.L^−1^; Yeast extract 20 g.L^−1^; Hemin 5 mL.L^−1^) and then used for DNA extraction as previously described [42]. For strain identification, we systematically performed a bifidobacteria PCR genus, a species multiplex PCR and partial sequencing of the 16S rRNA gene PCR product as previously described [43]. The specific identification of *B. animalis subsp. lactis* strains was performed as described by Kwon et al. (2005) [44].

### 2.3. Strain Genotyping

The clonal relationship of bifidobacteria belonging to the same species and isolated for a single individual was determined using pulsed field gel electrophoresis (PFGE) as previously described [45]. Bionumerics (Applied Math, Kortrijk, Belgium) was used to establish a similarity matrix for the DNA based on calculation of the Dice coefficient (pairwise comparison of strains). Then, dendrograms were generated with the UPGMA (unweighted pair group using arithmetic means) hierarchical algorithm. Strains sharing 80% of common bands were considered clonally related [46].

With PFGE being ineffective in achieving *B. animalis subsp. lactis* strains separation, strain-specific genotyping of *B. animalis subsp. lactis* was performed as described by Brinczinski et al. (2009) [47].

### 2.4. Acid and Bile Tolerance Assays

Testing tolerance to acid and bile has been considered to provide useful information for the evaluation of strains’ capabilities to endure the conditions of the gastrointestinal tract. The tolerance of strains to bile (simulated intestinal stress) and to acid and pepsin (simulated gastric stress) (except *animalis subsp. lactis* strains) was examined as previously described [43]. Tolerance to bile (0.3%) was evaluated after 5 h of incubation and tolerance to acid and pepsin was measured after 30 min of incubation. For stress tests, bifidobacteria viability was calculated as a percentage of survivors as compared to controls (culture without bile salts for intestinal stress and at pH 7 without pepsin for gastric stress). Strains were classified as tolerant or non-tolerant. Tolerant bifidobacteria were defined by a decrease in viability of less than 2 log_10_ CFU and non-tolerant bifidobacteria by a decrease in viability of more than 2 log_10_ CFU or no survival at all.

### 2.5. Tetracycline Resistance Characterization

Bifidobacteria have been reported to carry potential resistance genes to tetracycline [48]. Therefore, we investigated the minimum inhibitory concentration (MIC) of tetracycline using Etest^®^ tetracycline strips (bioMerieux, Marcy l’Etoile, France) as recommended by the manufacturer’s instructions. Clinically, resistance to tetracycline correspond to a MIC > 8 mg/L (CA-SFM/EUCAST 2021, https://www.sfm-microbiologie.org/2021/04/23/casfm-avril-2021-v1-0/, 9 Janaury 2022). Tetracycline genetic determinants responsible for tetracycline resistance among bifidobacteria are *tet* genes encoding ribosomal protection proteins, the most frequent gene being *tet*(W) [48]. Therefore, all strains were screened by PCR for the *tet*W gene using specific sense and antisense primers (Eurogentec), as previously described [48]. Alternatively, when necessary, degenerated primers targeting *tet*(M), *tet*(W), *tet*(O) and *tet*(32) were used as described previously [49].

## 3. Results

### 3.1. Characteristics of the Infants

A total of 32 infants (50% of male sex and 50% of female sex) were included in this study (Table S1). The neonates were born between 27 and 34 weeks of gestational age (53% by C-section) and their birth weight was comprised between 710 g and 2610 g. Majority of neonates received an antibiotherapy (72%) and were initially fed either formula for premature infants, or mixed fed (formula for premature infants + human milk). Only 8 of them were exclusively initially fed human milk (Table S1). Neonates were between 0.2 and 16.2 months of post-natal age at stool collection (Table 1).

### 3.2. Bifidobacteria Colonization

A total of 32 neonates displayed a bifidobacterial colonization profile (Table S1), and for 26 of them, at least two fecal samples at different sampling times (0.2 to 15.9 months of life) were obtained (Table 1). In this cohort, 11 pairs of twins were included (Table 1). In total, 60 fecal samples were analyzed.

The bifidobacteria counts ranged from 3.6 to 9.4 log_10_ CFU/g of feces, and 85 strains were isolated and identified at the species level as follows: *B. breve* (*n* = 31), *B. longum subsp. longum* (*n* = 32), *B. pseudocatenulatum* (*n* = 12), *B. bifidum* (*n* = 4), *B. animalis subsp. lactis* (*n* = 4), and *B. adolescentis* (*n* = 2). Each infant was colonized by either one (*n* = 15), two (*n* = 12), three (*n* = 4) or four different (*n* = 1) bifidobacteria species (Table 1). Additionally, we found that 20 infants harbored at less two strains of the same species at different sampling times (Table 1).

The majority of neonates initially received antibiotics and milk for premature infants or mixed feeding (Table S1). However, a two-sided Fisher exact test showed that there were no significant differences in each bifidobacteria species colonization among all infants (0.1 ≤ *p* ≤ 1, depending on the species).

### 3.3. Phenotypic Characterization of Bifidobacteria Strains

Eighty-one strains were tested for their tolerance towards intestinal stress (the four strains of *B. animalis subsp. lactis* species isolated were not tested because they are known to be resistant to intestinal stresses [47]).

The majority of the strains (*n* = 63) were not tolerant to bile or gastric stress. Thirteen (16%) strains (five *B. breve*, four *B. longum subsp. longum*, two *B. bifidum*, one *B. pseudocatenulatum* and one *B. adolescentis*) were tolerant to bile after 5 h of exposure. Five strains (6%; two *B. breve*, one *B. longum subsp. longum*, one *B. pseudocatenulatum*, and one *B. adolescentis*) were tolerant to gastric stress after 30 min of exposure (Table 2). Only 3 strains (two *B. breve* and one *B. adolescentis*) were resistant to both stress tests.

Eleven (13%) of the eighty-five strains were resistant to tetracycline (six *B. breve*, three *B. longum subsp. longum,* and two *B. pseudocatenulatum*; Table 3).

### 3.4. Genotypic Characterization and Relationships among Bifidobacteria Strains

**Figure 1:**
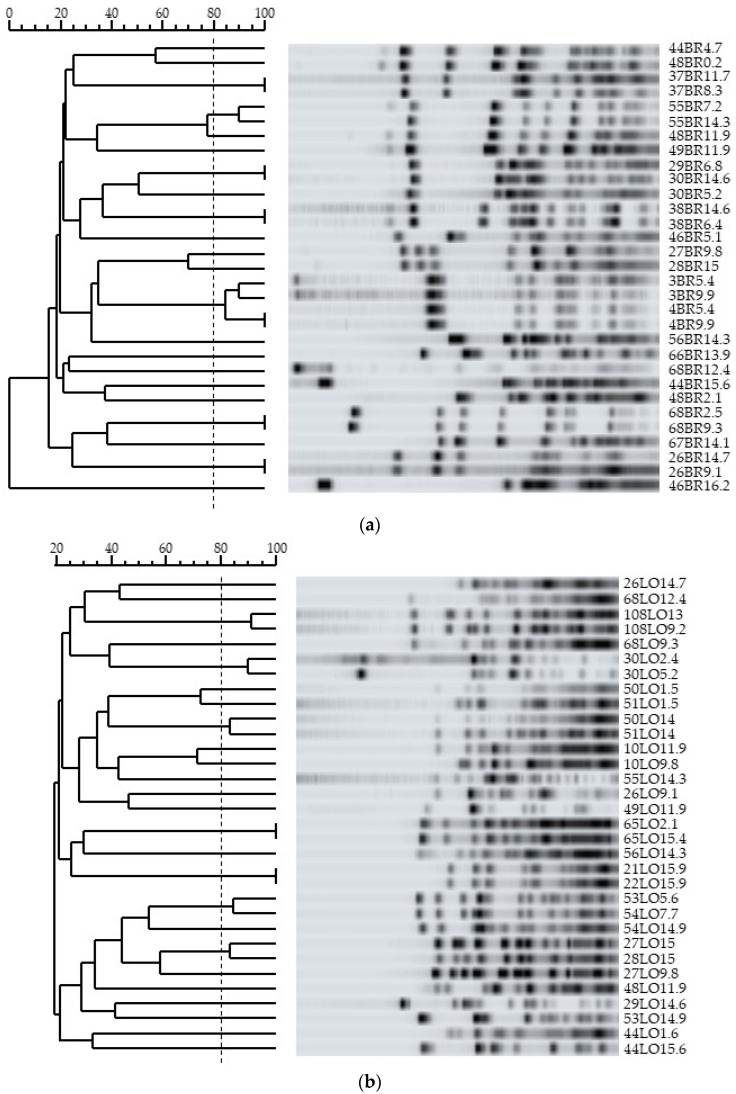

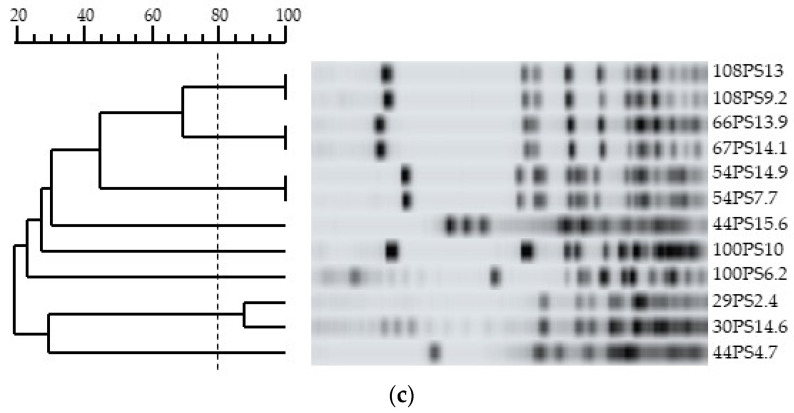
PFGE analysis of *B. breve* (**a**), *B. longum subsp. longum* (**b**) and *B. pseudocatenulatum* (**c**) strains. Clonally related strains share 80% of common bands (dotted line).

Fifty-seven different PFGE patterns (noted 1 to 57) were obtained from the eighty-one strains tested. Of the 31 *B. breve* strains, 32 *B. longum subsp. longum* strains and 12 *B. pseudocatenulatum* strains, 21, 25 and 8 different PFGE profiles were identified, respectively (Table 1 and Figure 1). One single profile was obtained for the two strains of *B. adolescentis* while the two strains of *B. bifidum* analyzed showed two different patterns. The four *B. animalis subsp. lactis* strains belonged to the same cluster 8.

Nine strains, including one susceptible to tetracycline (MIC ≤ 8 mg/L), were positive according to PCR for the *tet*(W) gene, and three strains had a PCR product amplified with degenerated primers targeting *tet*(M), *tet*(W), *tet*(O) and *tet*(32) (Table 3). Strains belonging to the same species with and without *tet* genes can coexist in the same infant (infants 13, 15, 28 and 29, Table 3). PFGE data suggest an absence of clonal relationship among these strains with and without *tet* genes and isolated from the same infant (Table 3). Therefore, these data suggest the absence of transfer of the tetracycline genes’ resistance among strains of the same species that can coexist in the same infant.

### 3.5. Bifidobacteria Population Dynamics during the First Year of Life

**Figure 2:**
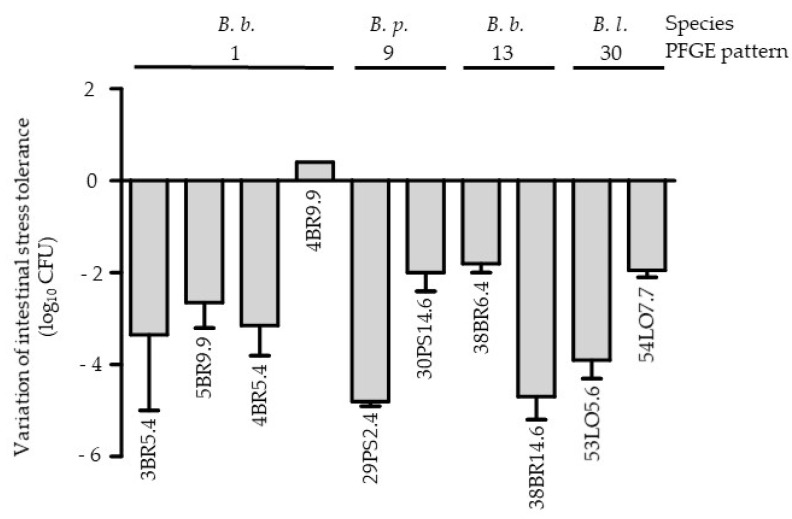
Comparison of the intestinal stress tolerance of bifidobacteria strains with same PFGE pattern. *B. b.*: *B. breve*, *B. p*.: *B. pseudocatenulatum*, *B. l.*: *B. longum subsp. longum*. The names of the strains are shown below each histogram. The errors bars represent the SEM deviation obtained with two independent experiments.

During the follow up, at least two fecal samples with bifidobacteria at different sampling times could be analyzed for 26 neonates (Table 1). In 20 neonates (77%), at least two strains of the same species were identified at different sampling times. Genotypic analysis of these strains showed that one individual could or could not harbor clonally related strains: clonally related *B. breve*, *B. longum subsp. longum* or *B. pseudocatenulatum* strains were only isolated for six, three, and two individuals, respectively (Table 1 and Figure 1). Interestingly, phenotypically, some of clonally linked strains (*n* = 10 including six *B. breve*, two *B. longum subsp. longum* and two *B. pseudocatenulatum*) showed different levels of tolerance towards intestinal stress (difference of more than 2 log_10_ of CFU; Figure 2). The PFGE data showed an absence of clonal relationships among strains with and without *tet* genes isolated from the same infant over time (Table 3).

### 3.6. Comparison of the Bifidobacteria Strains between Twins

Since our study was about premature neonates, we had a substantial number of samples from twins (11 pairs) and took the opportunity to compare the isolated bifidobacteria strains (Table 1). In these fecal samples, 18 *B. breve*, 20 *B. longum subsp. longum*, 6 *B. pseudocatenulatum*, 3 *B. animalis subsp. lactis*, 2 *B. bifidum* and 2 *B. adolescentis* were identified. Although twins had been initially fed formula or mix fed more frequently than singletons (*p* < 0.05, two-sided Fischer exact test), there were no significant differences in bifidobacteria species colonization between both singleton and twins (0.27 ≤ *p* ≤ 1, depending on the species, two-sided Fischer exact test).

When considering twins colonized with the same species at the same age, this included 9 (82%) pairs of twins and 26 strains (Table 4). Among these infants, seven pairs of twins also harbored at least one strain with the same PFGE pattern, suggesting that the majority of twins were colonized by clonally related strains (Table 4 and Figure 1). Only four pairs of twins showed one strain of *B. longum subsp. longum* or *B. breve* in each infant with different PFGE patterns corresponding to 38% of the strains isolated from these twins (Table 4). All strains of these nine were susceptible to tetracycline except for two strains (55BR14.3 and 56BR14.3) from twins 21 and 22, which were resistant (Table 4). Finally, the behavior of the bifidobacteria strains found in these twins toward intestinal and gastric stresses was equivalent in each neonate except for two pairs of twins (twins 1 and 2; twins 7 and 8), in whom strains showed different levels of tolerance in both twins (Table 4).

## 4. Discussion

In the present work, we described the longitudinal monitoring of the dominant bifidobacteria strains in neonates born preterm throughout the first year of life. Using bacterial culture allowing phenotypic and genotypic characterization of the strains, we showed that the dominant bifidobacteria composition of infant gut microbiota is not static but can vary over time in terms of species, strains, and bacterial properties of the strains.

Despite the interest in bifidobacteria, there are still discrepancies in terms of species distribution in the infant gut. If the most frequent species reported have previously been *B. longum*, *B. breve* and *B. bifidum* [50], in the present study we report other species, such as *B. adolescentis* and *B. peudocatenulatum*. *B. bifidum,* which is a species mostly associated with breastfed infants [51], was only isolated in three infants, including twins, in the present study. On the other hand, two infants (twins) were colonized by *B. adolescentis,* a species more commonly reported in adults’ guts than in infants’. Likewise, four neonates harbored *B. animalis subsp. lactis* species, all belonging to the same cluster, an adult-type species that is often a component of fermented milk products from the diet [52]. Our results demonstrated that there was a fluctuation in the intestinal dominant bifidobacteria at the species level in each infant, independently of the antibiotic intake or the initial feeding. Intra-species analysis using PFGE showed that some strains of the same species in the same infant are genetically unrelated suggesting that external factors may influence gut colonization. Indeed, we highlighted that some clonally related strains could have different biological responses towards intestinal stresses. This suggested that these strains, although genetically related, may have different phenotypes because of their gene expression profile due to their local environment stimulation. In favor of this hypothesis is the observation that the majority of clonally related strains observed in twins have the same phenotypic properties. With this longitudinal follow up and the sequential isolation of bifidobacteria strains, we showed that the similarities between bacteria species in twins are also found at the strains level and share the same phenotypic characteristics too. Our observations are in agreement with previous studies suggesting that the microbiota composition was predominantly shaped by non-genetic factors and that twins have more similar gut microbiota than unrelated infants [53,54]. However, the observation of strains stability we observed is in accordance with the limit of detection of differences among strains by the PFGE method. Indeed, PFGE has a limited resolution as comparison between strains is based on the presence or absence of DNA fragments. Further analysis through genome sequencing would be of interest to discern strain differences at a higher resolution using for instance single nucleotide polymorphisms.

We showed that the majority of bifidobacteria strains are not tolerant to intestinal and gastric stresses. As the study of gastric and intestinal stresses was performed in vitro, we cannot exclude the possibility that these strains would have behaved differently in vivo. However, in physiological conditions, bile acids are mainly absorbed by the ileum and do not reach the colon [55]. Moreover, the pH level in the small intestine and in the colon is higher than in the stomach [56]. Therefore, our results confirmed that bifidobacteria strains did not need to be tolerant to intestinal and gastric stresses to survive in the colon.

Altogether, our data suggest that we should not have a static view of the bifidobacteria composition of infant gut microbiota especially during the first year of life. We should rather take into account the possibility that strains of the same species could be different over time and have various phenotype and properties that reflect genes expression. Our study also demonstrates that the bifidobacteria of the gut of infants born preterm are not stable during the first year of life, and this creates the possibility for early-life interventional approaches to modify the gut microbiota of infants.

Tetracycline resistance is the most frequent resistance marker reported among *Bifidobacterium* species. In the present study, strain phenotypic characterization showed the existence of resistance to tetracycline among strains belonging to the same species and isolated from the same neonate. Therefore, we screened all strains for the presence or absence of *tet* genes. We showed that clonally related strains could either carry or not carry *tet* genes, suggesting an absence of horizontal transfer of the *tet* genes among strains co-inhabiting in the same environment. These data are in agreement with previous reports [48,57,58], and support our PFGE conclusions about the bacterial turnover of bifidobacteria at the species and strain level in the gut of PNs. Of note, one strain of our study (57BI10.8) was susceptible to tetracycline although the *tet*(W) gene was detected via PCR: we hypothesize that the *tet*(W) gene is not functional, as was previously reported [48]. Additionally, 13% of the bifidobacteria strains were resistant to tetracycline, which is a lower rate than the 28–33% rate previously reported [48,57,58].

In the present study, we chose to focus on the culture of bifidobacteria as this allowed us to characterize each isolate at the strain level, which is not yet possible via metagenomics that evaluates the potential genetic properties of bacteria [59]. Our data are original, as they fill a gap in the literature regarding bifidobacteria strains’ potential phenotypic and genetic properties that actually colonize the gut of infants.

## 5. Conclusions

To conclude, this work provides new insights into the in vivo dynamics of commensal bifidobacteria at the strain level in neonates born prematurely. Gut colonization by bifidobacteria species, some of which are associated with adult microbiota rather than neonates, is clearly instable during the first year of life. Moreover, phenotypic characteristics of the bifidobacteria strains are dependent on environmental pressure, as clonally related strains in singletons may have different phenotypes, but not in twins, which evolve in the same environment. In infants born preterm, whose gut microbiota is altered and are at risk of the origin of possible short- and long-term diseases, the promotion of health using probiotic and/or prebiotic supplementation is promising. Although the health benefits of bifidobacteria are widely accepted, the turnover of the intestinal bifidobacterial population may cast doubt on the link between specific bifidobacteria and host beneficial effects. The latter cannot be restricted to one species or to one strain, but at least to the association of different strains for one species. In this respect, our study supports the need for better basic knowledge of the strains found in intestinal microbiota in order to reliably investigate the impact of prebiotics and/or probiotics on the gut microbiota.

## Figures and Tables

**Table 1:** Dominant bifidobacteria isolated from feces of neonates born preterm.

Infants	Twins	Sampling Time (Months)	Strains	*Bifidobacterium* Species ^1^	Level of Colonization (log_10_ CFU.g^−1^ Feces)	PFGE Pattern
1	1; 2	5.4	3BR5.4	*breve*	9.4	1
		9.9	3BR9.9	*breve*	9.3	1
2	1; 2	5.4	4BR5.4	*breve*	6.5	1
		9.9	4BR9.9	*breve*	7.6	1
3		9.8	10LO9.8	*longum subsp. longum*	7.0	2
		11.9	10LO11.9	*longum subsp. longum*	8.5	3
4	4; 5	15.9	21LO15.9	*longum subsp. longum*	6.8	4
5	4; 5	15.9	22LO15.9	*longum subsp. longum*	7.3	4
6		9.1	26BR9.1	*breve*	9.3	5
		9.1	26LO9.1	*longum subsp. longum*	8.6	54
		14.7	26BR14.7	*breve*	7.8	5
		14.7	26LO14.7	*longum subsp. longum*	6.5	55
7	7; 8	9.8	27BR9.8	*breve*	7.9	6
		9.8	27LO9.8	*longum subsp. longum*	8.3	7
		15	27LO15	*longum subsp. longum*	8.0	8
8	7; 8	15	28BR15	*breve*	7.8	6
		15	28LO15	*longum subsp. longum*	9.1	8
9	9; 10	2.4	29PS2.4	*pseudocatenulatum*	8.5	9
		6.8	29BR6.8	*breve*	7.3	10
		14.6	29LO14.6	*longum subsp. longum*	7.6	56
10	9; 10	2.4	30LO2.4	*longum subsp. longum*	8.2	57
		5.2	30LO5.2	*longum subsp. longum*	8.5	57
		5.2	30BR5.2	*breve*	7.9	11
		14.6	30BR14.6	*breve*	8.8	10
		14.6	30PS14.6	*pseudocatenulatum*	6.3	9
11		8.3	37BR8.3	*breve*	8.5	12
		11.1	37BR11.1	*breve*	9.4	12
12		6.4	38BR6.4	*breve*	5.3	13
		14.6	38BR14.6	*breve*	8.8	13
		14.6	38LA14.6	*animalis subsp. lactis*	7.8	ND
13		1.6	44LO1.6	*longum subsp. longum*	8	14
		4.7	44PS4.7	*pseudocatenulatum*	8.3	15
		4.7	44BI4.7	*bifidum*	8.5	ND
		4.7	44BR4.7	*breve*	8.7	16
		15.6	44LO15.6	*longum subsp. longum*	6.2	18
		15.6	44PS15.6	*pseudocatenulatum*	5.8	19
		15.6	44BR15.6	*breve*	9.0	17
		15.6	44BI15.6	*bifidum*	7.3	ND
14		5.1	46BR5.1	*breve*	8.5	20
		16.2	46BR16.2	*breve*	3.9	21
15	15; 16	0.2	48BR0.2	*breve*	6.6	22
		2.1	48BR2.1	*breve*	7.2	23
		11.9	48BR11.9	*breve*	8.0	24
		11.9	48LO11.9	*longum subsp. longum*	6.6	25
16	15; 16	11.9	49BR11.9	*breve*	8.5	24
		11.9	49LO11.9	*longum subsp. longum*	8.6	26
17	17; 18	1.5	50LO1.5	*longum subsp. longum*	8.6	27
		14	50LO14	*longum subsp. longum*	8.5	28
18	17; 18	1.5	51LO1.5	*longum subsp. longum*	9.0	29
		14	51LO14	*longum subsp. longum*	8.8	28
		14	51LA14	*animalis subsp. lactis*	ND	ND
19	19; 20	5.6	53LO5.6	*longum subsp. longum*	7.4	30
		14.9	53LO14.9	*longum subsp. longum*	8.3	31
20	19; 20	7.7	54LO7.7	*longum subsp. longum*	7.6	30
		7.7	54PS7.7	*pseudocatenulatum*	3.6	32
		14.9	54LO14.9	*longum subsp. longum*	9.3	33
		14.9	54PS14.9	*pseudocatenulatum*	5.6	32
21	21; 22	7.2	55BR7.2	*breve*	9.3	34
		14.3	55BR14.3	*breve*	7.6	35
		14.3	55LO14.3	*longum subsp. longum*	8.6	53
22	21; 22	14.3	56BR14.3	*breve*	7.9	36
		14.3	56LO14.3	*longum subsp. longum*	8.6	37
23	23; 24	10.8	57BI10.8	*bifidum*	8.5	38
24	23; 24	15.3	58BI15.3	*bifidum*	8.3	39
25		2.1	65LO2.1	*longum subsp. longum*	9.3	40
		15.4	65LO15.4	*longum subsp. longum*	7.3	40
26	26; 27	2.4	66LA2.4	*animalis subsp. lactis*	5.3	ND
		13.9	66BR13.9	*breve*	8.7	41
		13.9	66PS13.9	*pseudocatenulatum*	9.0	42
27	26; 27	2,4	67LA2.4	*animalis subsp. lactis*	3.3	ND
		14.1	67BR14.1	*breve*	8.1	43
		14.1	67PS14.1	*pseudocatenulatum*	8.1	42
28		2.5	68BR2.5	*breve*	9.0	44
		9.3	68BR9.3	*breve*	8.8	44
		9.3	68LO9.3	*longum subsp. longum*	7.8	45
		12.4	68BR12.4	*breve*	8.1	47
		12.4	68LO12.4	*longum subsp. longum*	6.5	46
29		6.2	100PS6.2	*pseudocatenulatum*	7.3	48
		10	100PS10	*pseudocatenulatum*	9.2	49
30	30; 31	2.2	101AD2.2	*adolescentis*	3.6	50
31	30; 31	2.2	102AD2.2	*adolescentis*	7.7	50
32		9.2	108LO9.2	*longum subsp. longum*	6.9	52
		9.2	108PS9.2	*pseudocatenulatum*	8.8	51
		13	108LO13	*longum subsp. longum*	7.8	52
		13	108PS13	*pseudocatenulatum*	9.0	51

Out of 59 fecal samples from 32 neonates analyzed, 85 bifidobacteria strains were isolated. ^1^ Species found per sample base on 10 randomly chosen colonies; ND: not determinate.

**Table 2:** Tolerant bifidobacteria strains towards intestinal and gastric stresses.

Intestinal Stress			Gastric Stress		
Infants	Strains	Species	Infants	Strains	Species
2	4BR9.9	*breve*	13	44LO1.6	*longum subsp. longum*
7	27LO15	*longum subsp. longum*	13	44PS15.6	*pseudocatenulatum*
9	29LO14.6	*longum subsp. longum*	26	66BR13.9	*breve*
10	30PS14.6	*pseudocatenulatum*	28	68BR12.4	*breve*
12	38BR6.4	*breve*	30	101AD2.2	*adolescentis*
13	44BI4.7	*bifidum*			
13	44BI15.6	*bifidum*			
13	44BR4.7	*breve*			
20	54LO7.7	*longum subsp. longum*			
26	66BR13.9	*breve*			
28	68LO12.4	*longum subsp. longum*			
28	68BR12.4	*breve*			
30	101AD2.2	*adolescentis*			

Tolerant bifidobacteria showed a viability decreased by <2 log_10_ CFU and non-tolerant bifidobacteria showed a viability decreased by >2 log_10_ CFU or no survival at all. Strains tolerant to intestinal and gastric stresses are highlighted in gray.

**Table 3:** Bifidobacteria isolated from the same infant with and without *tet* genes.

Infant	Strains	Species	*tet* Genes	Tetracycline MIC (mg/L)	Tetracycline Sensitivity	PFGE Pattern
13	44LO1.6	*longum subsp. longum*	-	1	S	14
	44PS4.7	*pseudocatenulatum*	-	1.5	S	15
	44BI4.7	*bifidum*	-	1.5	S	ND
	44BR4.7	*breve*	-	1	S	16
	44LO15.6	*longum subsp. longum*	-	1	S	18
	44PS15.6	*pseudocatenulatum*	+	32	R	19
	44BI15.6	*bifidum*	-	1.5	S	ND
	44BR15.6	*breve*	-	1.5	S	17
15	48BR0.2	*breve*	-	0.75	S	22
	48BR2.1	*breve*	+	16	R	23
	48BR11.9	*breve*	-	1.5	S	24
	48LO11.9	*longum subsp. longum*	-	0.5	S	25
21 *	55BR7.2	*breve*	+	16	R	34
	55BR14.3	*breve*	+	16	R	35
	55LO14.3	*longum subsp. longum*	-	0.5	S	53
22 *	56BR14.3	*breve*	+	16	R	35
	56LO14.3	*longum subsp. longum*	-	0.75	S	36
23	57BI10.8	*bifidum*	+	1.5	S	37
28	68BR2.5	*breve*	+	24	R	43
	68BR9.3	*breve*	+	24	R	44
	68LO9.3	*longum subsp. longum*	+	48	R	45
	68BR12.4	*breve*	-	1.5	S	47
	68LO12.4	*longum subsp. longum*	-	0.5	S	46
29	100PS6.2	*pseudocatenulatum*	-	1.5	S	48
	100PS10	*pseudocatenulatum*	+	8	R	49
32	108LO9.2	*longum subsp. longum*	+	8	R	52
	108PS9.2	*pseudocatenulatum*	-	1.5	S	51
	108LO13	*longum subsp. longum*	+	12	R	52
	108PS13	*pseudocatenulatum*	-	1	S	51

MIC: minimum inhibitory concentration; R: resistant (MIC > 8 mg/L); S: susceptible, ND: not determinate, +: presence, -: absence. *: twins. Strains with *tet* genes are highlighted in gray.

**Table 4:** Characteristics of strains found in feces of twins with same species at the same age.

Twins	Sampling Time (Months)	Species	PFGE Pattern	Tetracycline Susceptibility	Intestinal Stress	Gastric Stress
1 and 2	5.4	*breve*	1	S	NT	NT
	9.9	*breve*	1	S	NT and T	NT
4 and 5	15.9	*longum subsp. longum*	4	S	NT	NT
7 and 8	15	*longum subsp. longum*	8	S	T and NT	NT
15 and 16	11.9	*breve*	24	S	NT	NT
		*longum subsp. longum*	25 and 26	S	NT	NT
17 and 18	1.5 14	*longum subsp. longum longum subsp. longum*	27 and 29 28	S S	NT NT	NT NT
19 and 20	14.9	*longum subsp. longum*	31 and 33	S	NT	NT
21 and 22	14.3	*breve*	35	R	NT	NT
		*longum subsp. longum*	53 and 36	S	NT	NT
26 and 27	2.4	*animalis subsp. lactis*	ND	ND	ND	ND
30 and 31	2.2	*adolescentis*	50	S	T and NT	T and NT

ND: not determinate; S: sensitive; R: resistant; NT: non tolerant; T: tolerant. Both *B. animalis subsp. lactis* strains (twins 21 and 22) belong to cluster 8 according to the sequence-based typing method.

## Data Availability

Not applicable.

## Supplementary Materials

The following supporting information can be downloaded at: https://www.mdpi.com/article/10.3390/microorganisms10030654/s1, Table S1: Characteristics of the cohort.
