# Supplementary material for: Isolation and Characterization of Commensal Bifidobacteria Strains in Gut Microbiota of Neonates Born Preterm: A Prospective Longitudinal Study
Source: Microorganisms. 2022 Mar 18;10(3):654. doi: 10.3390/microorganisms10030654 (PMC8951322; doi:10.3390/microorganisms10030654)
Supplement: Supplementary file 1 [file microorganisms-10-00654-s001.zip › microorganisms-1570881-supplementary.pdf]

Table S1: Characteristics of the cohort

Infants	Sex	Birth weight (g)	Gestational age (weeks)	Pregnancy	Delivery mode	*Per partum* antibiotic therapy	Neonate antibiotic therapy [1]	Initial feeding
1	Male	1030	28	Twin	Vaginal	Yes	Yes	?
2	Male	1000	28					
3	Female	1120	27	Singleton	Vaginal	Yes	Yes	HM
4	Male	1620	30	Twin	Cesarean	No	No	HM
5	Female	1530	30					Mix
6	Female	1510	29	Singleton	Vaginal	No	Yes	HM
7	Female	1670	32	Twin	Cesarean	Yes	Yes	PM
8	Female	2170	32					
9	Male	1590	31	Twin	Cesarean	Yes	Yes	PM
10	Male	1685	31					
11	Male	815	30	Singleton	Cesarean	No	Yes	HM
12	Male	710	31	Singleton	Cesarean	No	Yes	HM
13	Male	1730	34	Singleton	Cesarean	No	No	?
14	Male	2160	33	Singleton	Vaginal	Yes	No	PM
15	Male	2420	33	Twin	Vaginal	Yes	No	PM
16	Male	2610	33					
17	Male	1960	34	Twin	Cesarean	No	No	Mix
18	Male	1420	34				Yes	PM
19	Female	1690	32	Twin	Vaginal	Yes	No	PM
20	Female	1750	32				Yes	
21	Female	2000	34	Twin	Cesarean	Yes	Yes	PM
22	Female	1430	34					
23	Female	1870	33	Twin	Vaginal	Yes	Yes	Mix
24	Female	1500	33					
25	Female	2400	34	Singleton	Vaginal	Yes	Yes	?
26	Male	2010	33	Twin	Vaginal	Yes	Yes	Mix
27	Female	2080	33					PM
28	Male	1880	33	Singleton	Vaginal	No	Yes	?
29	Female	1260	30	Singleton	Cesarean	?	No	HM
30	Female	1340	31	Twin	Cesarean	Yes	Yes	HM
31	Female	1000	31					
32	Male	1970	31	Singleton	Cesarean	Yes	Yes	Mix

[1]: before 30 days of life; ?: missing data; HM: human milk; PM: milk for premature infant; Mix: HM + PM
